# Computational modeling of blood component transport related to coronary artery thrombosis in Kawasaki disease

**DOI:** 10.1371/journal.pcbi.1009331

**Published:** 2021-09-07

**Authors:** Noelia Grande Gutiérrez, Mark Alber, Andrew M. Kahn, Jane C. Burns, Mathew Mathew, Brian W. McCrindle, Alison L. Marsden

**Affiliations:** 1 Department of Mechanical Engineering, Stanford University, Stanford, California, United States of America; 2 Department of Mathematics and Interdisciplinary Center for Quantitative Modeling in Biology, University of California, Riverside, Riverside, California, United States of America; 3 Department of Medicine, University of California, San Diego, San Diego, California, United States of America; 4 Department of Pediatrics, University of California, San Diego, San Diego, California, United States of America; 5 The Hospital for Sick Children, University of Toronto, Toronto, Ontario, Canada; 6 Department of Pediatrics, Bioengineering and Institute for Computational and Mathematical Engineering, Stanford University, Stanford, California, United States of America; University of Virginia, UNITED STATES

## Abstract

Coronary artery thrombosis is the major risk associated with Kawasaki disease (KD). Long-term management of KD patients with persistent aneurysms requires a thrombotic risk assessment and clinical decisions regarding the administration of anticoagulation therapy. Computational fluid dynamics has demonstrated that abnormal KD coronary artery hemodynamics can be associated with thrombosis. However, the underlying mechanisms of clot formation are not yet fully understood. Here we present a new model incorporating data from patient-specific simulated velocity fields to track platelet activation and accumulation. We use a system of Reaction-Advection-Diffusion equations solved with a stabilized finite element method to describe the evolution of non-activated platelets and activated platelet concentrations [AP], local concentrations of adenosine diphosphate (ADP) and poly-phosphate (PolyP). The activation of platelets is modeled as a function of shear-rate exposure and local concentration of agonists. We compared the distribution of activated platelets in a healthy coronary case and six cases with coronary artery aneurysms caused by KD, including three with confirmed thrombosis. Results show spatial correlation between regions of higher concentration of activated platelets and the reported location of the clot, suggesting predictive capabilities of this model towards identifying regions at high risk for thrombosis. Also, the concentration levels of ADP and PolyP in cases with confirmed thrombosis are higher than the reported critical values associated with platelet aggregation (ADP) and activation of the intrinsic coagulation pathway (PolyP). These findings suggest the potential initiation of a coagulation pathway even in the absence of an extrinsic factor. Finally, computational simulations show that in regions of flow stagnation, biochemical activation, as a result of local agonist concentration, is dominant. Identifying the leading factors to a pro-coagulant environment in each case—mechanical or biochemical—could help define improved strategies for thrombosis prevention tailored for each patient.

## Author Summary

Computational studies aiming to model thrombosis often rely on an arterial wall injury. Collagen and other extracellular matrix components are exposed to the bloodstream, which facilitates platelet adhesion to the wall and subsequent clot formation. However, these models are not adequate to explain thrombosis in other settings where even in the absence of a focal lesion, clots may still form under certain flow conditions. Coronary artery aneurysm thrombosis following KD is an example of the need to understand the mechanisms of thrombus initiation in the absence of an extrinsic factor. This study provides a new framework to investigate thrombus initiation in KD from a patient-specific perspective, which integrates fluid mechanics and biochemistry and which could help quantify the pro-coagulant environment induced by the aneurysm and become a predictive tool. The work presented here has broad relevance to other clinical situations where flow stagnation and transport are driving factors in thrombus formation.

## Introduction

Coronary artery thrombosis is the major risk associated with Kawasaki disease (KD) [1]. Long-term management of KD patients with persistent aneurysms requires thrombotic risk assessment and clinical decisions regarding the administration of anticoagulation therapy [1]. Typically, medication prescribed to these patients falls into two main categories of anticoagulants: vitamin K inhibitors such as Warfarin, acting on coagulation factors, and antiplatelet drugs such as Clopidogrel, acting on the P2Y receptor on the platelet surface related to Adenosine Diphosphate (ADP) mediated platelet activation. Also, oral Xa direct inhibitors or direct thrombin inhibitors may be used as an alternative anticoagulation therapy in KD [1]. These are currently not approved for pediatric use, although clinical trials are on-going [2]. Patient-specific computational modeling has provided new insight regarding thrombotic risk for KD patients [3–6], demonstrating that hemodynamic variables obtained from computational fluid dynamics (CFD) simulations have the potential to identify regions prone to thrombosis [3]. However, the underlying mechanisms of clot formation, which could help determine favorable strategies for thrombosis prevention, are not fully understood.

**Fig 1:**
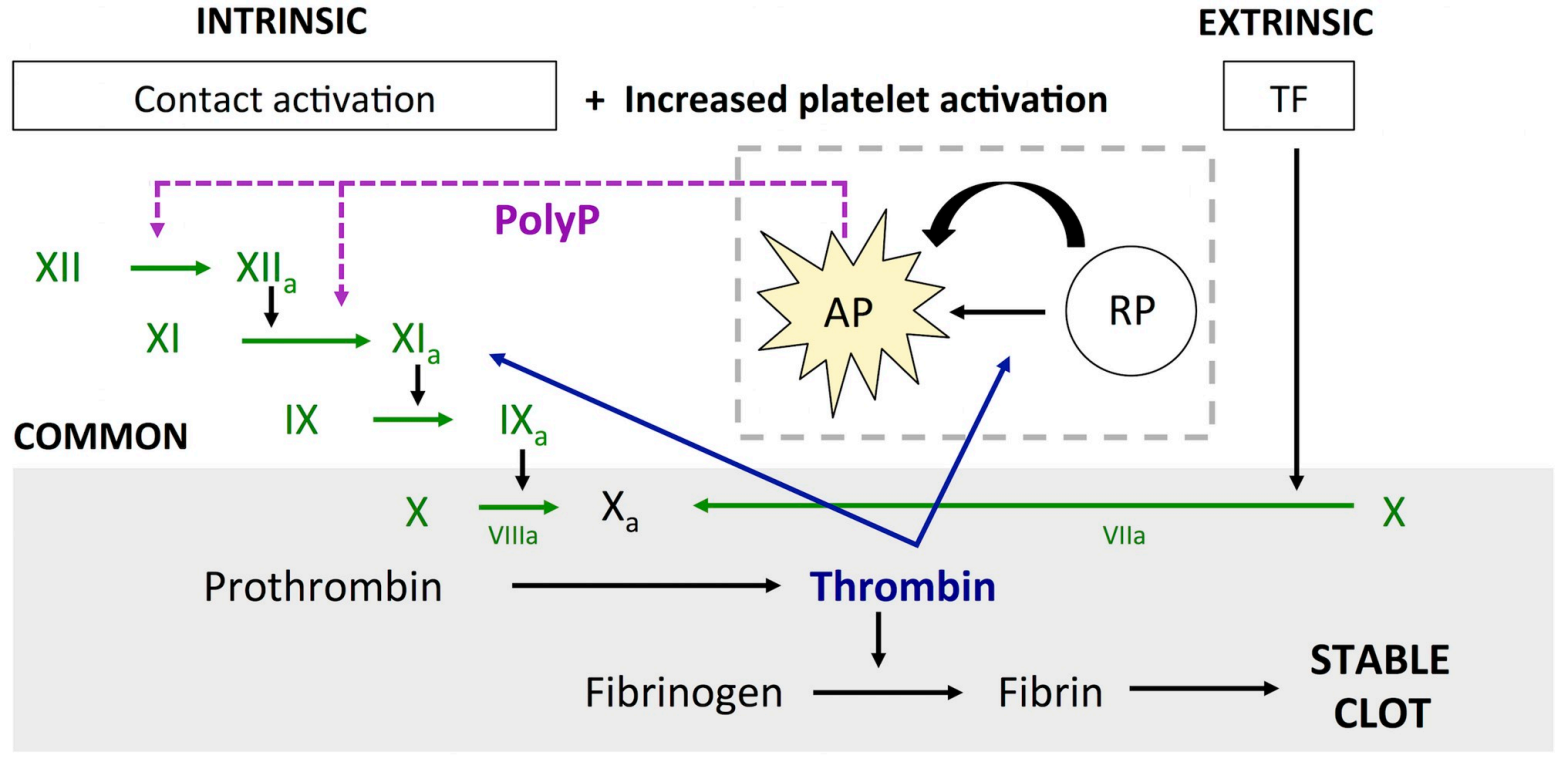
Simplified representation of the coagulation cascade. Intrinsic and extrinsic pathways and proposed pathway for aneurysm induced thrombosis (increased platelet activation + PolyP). TF = Tissue Factor, AP = Activated platelet, RP = Resting platelet, PolyP = Polyphosphate.

The coagulation cascade has been widely studied. In particular, in the setting of hemostasis. The two coagulation pathways, the intrinsic or contact pathway and the extrinsic pathway initiated by tissue factor (TF), converge to the common pathway leading to activation of factor X and generation of thrombin, fibrin, and ultimately a stable clot (Fig 1). Platelets play an essential role in thrombus formation, especially at early stages, and can be activated by thrombin and through interactions with collagen and von Willebrand factor (vWF). Upon activation, platelets undergo changes in their membrane that facilitate adhesion to the vessel wall and aggregation to other platelets, and release the content stored in their dense granules. Chemical agonists released from platelet dense granules include adenosine diphosphate (ADP), thromboxane A2 (TXA2), which are involved in platelet activation; and polyphosphate (PolyP) [7] reported in recent studies as a potential activator for the intrinsic coagulation pathway [8–11].

Computational studies aiming to model arterial thrombosis often rely on an arterial wall injury that exposes collagen, vWF, and other sub-endothelial components to the bloodstream, which facilitates platelet adhesion to the wall even in a non-activated state [12], and subsequent clot formation. Also, extravascular TF can come in contact with the bloodstream providing the trigger required to initiate the extrinsic coagulation cascade and generate thrombin. Several studies have modeled the extrinsic coagulation pathway and most of the chemical reactions involved using computational methods. Models by Fogelson and Kuharsky et al. [13–15] provide a comprehensive description of the coagulation cascade, including the interactions of coagulation factors, platelet deposition and aggregation; and flow-mediated transport to explore the role of hemodynamics on early coagulation events. Work by Leiderman et al. [16, 17] investigated the implications of blood components transport in thrombus growth. Later studies further demonstrated the essential role that transport physics plays in thrombosis [18, 19], in particular during clot formation and growth. Work by Chatterjee et al. [20] proposed a systems biology approach to model coagulation initiation that also considered the intrinsic coagulation pathway and the interplay between the extrinsic and intrinsic coagulation pathways. However, these models are not adequate to explain thrombosis in other settings where even in the absence of wall injury, blood clots may still form under complex flow conditions. Experimental data suggest the role of hemodynamics in thrombus initiation and growth is two-fold: shear forces can contribute to platelet activation, and flow stagnation facilitates platelet aggregation, driving thrombus formation and growth [21]. Also, under elevated shear conditions shear-induced platelet aggregation can lead to occlusive arterial thrombosis [12, 22]. This process is mediated by vWF and is independent of platelet activation. In addition, platelets can be activated by either mechanical (shear-induced platelet activation) or biochemical stimuli, which suggest different potential targets for thromboprophylaxis [15]. Data from both experimental [23, 24] and computational studies [25, 26] show that platelet activation depends not only on the level of shear rate exposure but also on the length of the exposure, suggesting that longer exposure to moderate shear levels could also induce platelets change to an activated state. Platelet activation potential and shear rate exposure have been investigated related to stenosis in coronary or carotid arteries, as a means to quantify the effect of shear rate on platelet activation [23, 25, 27, 28], and regarding high shear flows induced by medical devices [24, 29, 30]. Prior CFD studies have considered the effects of shear-induced platelet activation by using activation functions that rely on critical thresholds for shear stress, either low [28] or high [31]. Work by Di Achille et al. [32] used Lagrangian particle tracking to obtain information on the shear history of particles traveling through the domain. In this case, shear history was used to compute a platelet activation potential index, which was proposed as a predictor for areas prone to intraluminal thrombosis in abdominal aortic aneurysms. Other models, focused on the TF or extrinsic coagulation pathway considered platelet activation as a function of chemical agonists and thrombin concentrations and proposed a thrombus growth model, based on platelet-platelet and platelet-wall adhesion forces, at low and high shear rate levels [33].

Typically thrombosis models have been coupled with the flow, widely using a 2D approach, relying on idealized flow waveforms [16, 34–38] and considering small computational domains on the order of micrometers, localized in the proximity of a TF source. A few recent studies have presented 3D models to investigate low shear and high shear thrombosis yet the majority have considered idealized rather than patient-specific anatomies [22, 33, 39, 40]. Yadzani et al. [41] proposed a hemodynamic transport model to predict the location and extent of thrombus in aortic dissection in mice. Regarding the use of patient-specific anatomies in the context of thrombosis modeling, work by Di Achille et al. [32, 42] and Arzani et al. [43] proposed hemodynamic based metrics to identify regions prone to thrombosis in abdominal aortic aneurysms. However, these studies considered only a mechanical perspective of thrombus formation and did not consider the biochemical aspects related to thrombus initiation. Menichini et al. [28] presented a computational model to predict false lumen thrombosis in patient-specific aortic dissection anatomies, based on fluid shear rate, residence time, and platelet distribution. More recently, Li et al. [44] presented a predictive model for intraluminal thrombus formation in diabetic retinal microaneurysms, which combines a continuum and a particle model to simulate platelet aggregation on thrombogenic surfaces inside these microaneurysms. Our previous work in KD patients [3] also provided wall shear stress exposure and residence time as metrics for thrombotic risk stratification.

One limitation of current thrombosis models is their reliance on assumptions about the thrombotic potential of the wall, for which there is very limited quantitative data. These models require that initial extrinsic factor, TF, or assumed collagen or vWF concentration is prescribed as the initiator for the coagulation cascade. Although these assumptions hold in the case of trauma or athero-thrombosis, there are other scenarios where no data on the thrombogenic potential of the arterial wall is available. Therefore, these models fail to accurately represent the process of clot formation in such cases. Coronary artery aneurysm thrombosis following KD is an example of the need to understand the mechanisms of thrombus initiation in the absence of an extrinsic factor. No obvious wall injury, which would enable a local release of TF, has been reported concerning thrombosis in KD aneurysms. Therefore, we hypothesized that the intrinsic or contact coagulation pathway may be responsible for thrombus formation in this setting. Our previous studies related to KD aneurysms, have related thrombosis risk with abnormal hemodynamics, suggesting that hemodynamics are likely the primary driver. We present a new model that incorporates information from patient-specific simulated velocity fields to track platelet activation and accumulation in patient-specific coronary artery aneurysms caused by KD. Increased concentration of activated platelets and agonists induces a pro-coagulant environment that may drive thrombus initiation and growth. This hypothesis is based on recent studies that have suggested that critical concentration of PolyP, released from platelets upon activation, can activate factor XII and initiate the intrinsic coagulation cascade [7–10] and data that relates critical concentrations of ADP with platelet activation and aggregation [45]. The main goal of this study is to investigate the potential for platelet activation from patient-specific hemodynamics and establish links between platelet activation—mechanical and biochemical—and initiation of the intrinsic coagulation pathway.

## Methods

### Ethics statement

This study was approved by the Institutional Review Board at Stanford University and The Hospital for Sick Children, University of Toronto. Written subject consent or assent and parental written consent were obtained as appropriate for the imaging and simulation studies.

### Patient population

We considered six KD patients with aneurysms and one control patient with normal coronary arteries. The diseased group was drawn from our previous study on patient-specific hemodynamics and risk of thrombosis in KD [3]. This group included three patients classified as low risk for thrombosis according to all the hemodynamic metrics analyzed in [3], and three patients with confirmed thrombosis, featuring different aneurysms shapes, sizes, and locations.

### Shear induced platelet activation: Cumulative shear rate

To quantify cumulative shear rate exposure in the 3D domain we used a Lagrangian approach, computing shear rate exposure along path lines calculated from a patient-specific velocity field. Shear rate is defined as:

$$\begin{array}{c} SR=\sqrt{2E:E}\;, \end{array}$$ (1)

where *E* is the rate of strain tensor

$$\begin{array}{c} E=\frac{1}{2}\left(\nabla u+\nabla u^{T}\right)\;. \end{array}$$ (2)

Cumulative shear rate was defined as a linear function of shear rate exposure time

$$\begin{array}{c} SR_{cum}=\sum SR\cdot \Delta t\;. \end{array}$$ (3)

**Fig 2:**
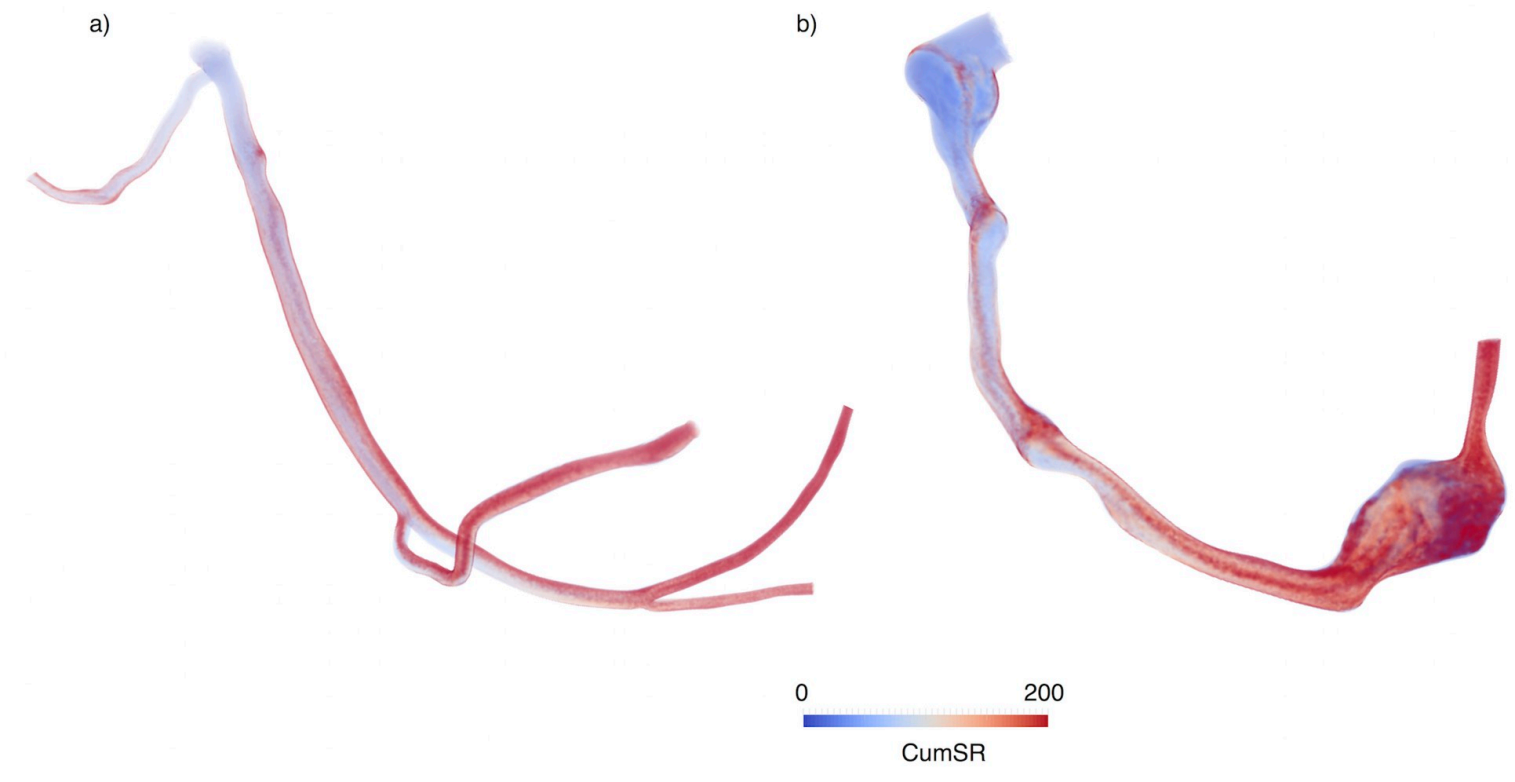
Cumulative shear rate distribution in Kawasaki disease right coronary artery. Normal coronary vs. coronary artery aneurysm. CumSR = Cumulative Shear rate.

**Fig 3:**
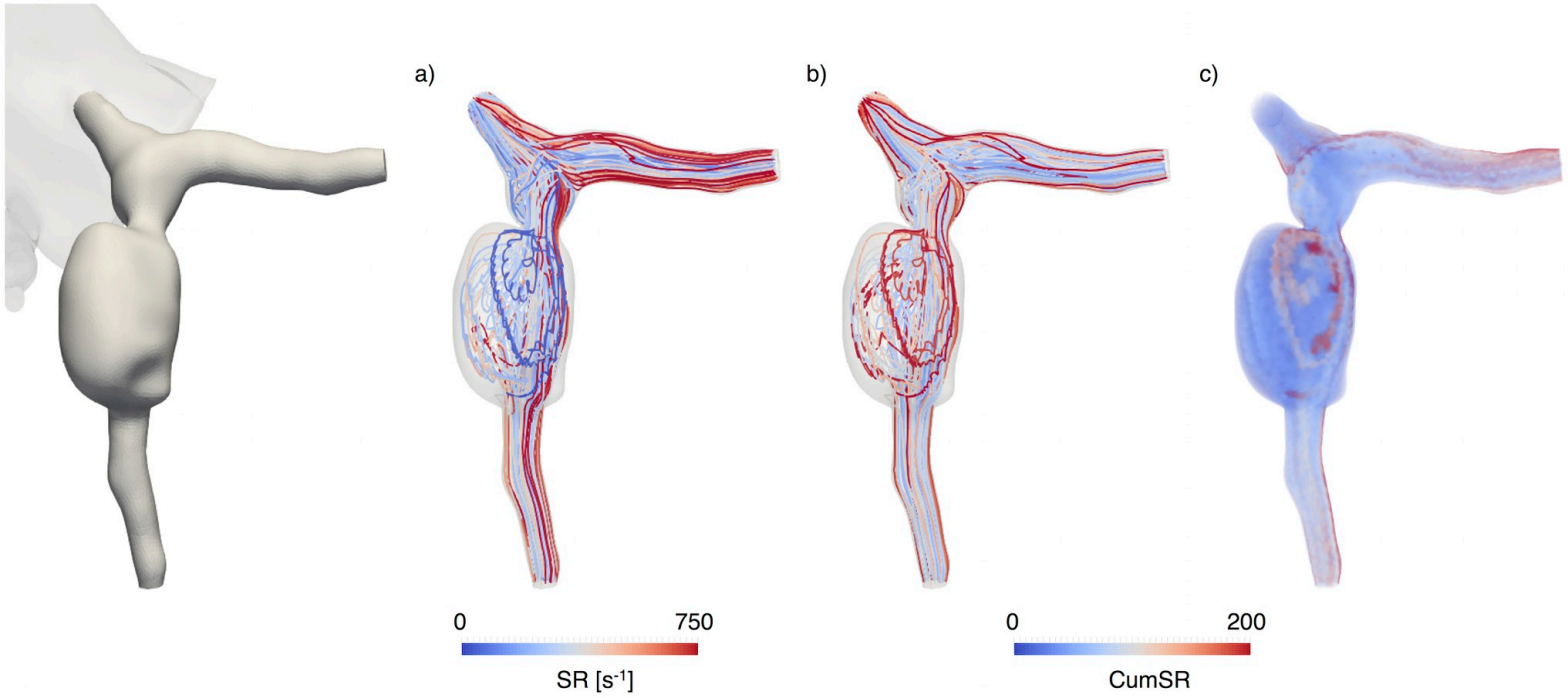
Path lines of particles released at the beginning of the cardiac cycle in a left coronary branch (P4). a) Shear Rate (SR); b) Cumulative shear rate (CumSR) along path lines; c) Cumulative shear rate 3D distribution (CumSR), computed as a time average for the cardiac cycle.

To initiate the calculation, massless particles representing platelets (Stokes number ≪ 1) were released at the most proximal section of the coronary artery model (ostium) and their trajectories were time-integrated using a fourth order Runge-Kutta scheme based on the velocity field obtained from a patient-specific blood flow simulation [3]. We obtained hemodynamic quantities, including instantaneous or cumulative shear rate, along these path lines. Considering a sufficient number of seeding points at the inlet, we were able to retrieve a full 3D reconstruction of the average cumulative shear rate. This 3D field represents the average cumulative shear rate that a platelet, at a specific location in the 3D model, would be exposed to, as it travels along the coronary artery (Fig 2). The number of seeds was determined to match the number of nodes from the finite element mesh at the inlet for each model, therefore the number of seeds was adjusted to the size of the domain for each case. The simulation was initiated with the release of the particles at the seed points (representing platelets entering the domain) and ended when one of the following was achieved: path line reached the distal outlet (platelet leaving the coronary model), stagnation (constant point coordinates of the path line for more than one cardiac cycle), the maximum number of cycles prescribed for the simulation was reached. For this study, we set the maximum number of cardiac cycles for the simulation to 30 cycles, based on previous work [3]. Path lines were calculated in parallel by distributing the number of seed points to multiple processors so that the integration of each path line was performed independently. The cumulative shear rate computed for each trajectory was combined to obtain an average 3D cumulative shear rate field in a post-processing step. Examples of the cumulative shear rate distributions for a normal right coronary artery and a right coronary artery with an aneurysm are presented in Fig 2. The cumulative shear rate in the normal coronary artery increases linearly as a function of distance from the ostium, as expected from the definition (Eq 3). On the other hand, in the aneurysm case, we observe a non-linear distribution, with regions of high cumulative shear rate in proximal and medial regions of the coronary artery. Visualization of the path lines (Fig 3) shows the contrast between high shear rates and high cumulative shear rates in different regions of the model.

To describe platelet activation related to high cumulative shear rate, we used an activation function, based on the cumulative shear rate levels observed in the healthy coronary model, and reported literature values for platelet activation indices found in regions prone to thrombosis [23, 25]. The activation function was defined as:

$$\begin{array}{c} A\left(SR\right)=k_{AP}^{SR}f\left(SR_{cum}\right)\;, \end{array}$$ (4)

where

$$\begin{array}{c} f\left(SR_{cum}\right)=\{\begin{array}{cc} 0 & SR_{cum}\leq 250\\ \frac{SR_{cum}-250}{250} & 250<SR_{cum}\leq 500\\ 1 & 500<SR_{cum}\;, \end{array} \end{array}$$ (5)

and $k_{AP}^{SR}$ is the rate for platelet activation due to cumulative shear rate.

We chose four time points equally distributed along the cardiac cycle for particles release to compute path lines (0, 0.25T, 0.5T, 0.75T, where T is the period of the cardiac cycle). Although we observed differences in cumulative shear rate distribution when comparing results from particles released at the beginning of the cardiac cycle to other time points, we did not observe substantial differences when increasing the number of time points up to ten.

### Reaction-Advection-Diffusion equations

We use a continuum approach to model the transport of the main blood components and chemical agonists involved in thrombus initiation based on the models presented by Ananad [31] and Leiderman and Fogelson [16]. We solve the Reaction-Advection-Diffusion (RAD) equations using a stabilized Finite Element Method suitable for advection dominated problems [46], to compute the temporal evolution of the concentrations of non-activated platelets [*RP*] and activated platelets [*AP*], and local concentrations of Adenosine Diphosphate [*ADP*] and PolyPhosphate [*PolyP*]. To perform this simulation, sub-models of the right and left coronary branches were considered independently. Activation of platelets is modeled considering two different contributions to the reaction term: mechanical activation, a function of cumulative shear rate, and biochemical activation, a function of the local concentration of activated platelets and agonists such as ADP. We define the advection-diffusion operator as:

$$\begin{array}{c} L\left(A_{i}\right):=\frac{\partial A_{i}}{\partial t}+\nabla \cdot \left(uA_{i}\right)-D_{A_{i}}\nabla^{2}A_{i}\;, \end{array}$$ (6)

where [*A*_*i*_] represents the concentration of the i^*th*^ chemical specie considered and **u** is the patient-specific pre-computed fluid velocity. The velocity fields were computed using Simvascular [47] as described in our previous work [3]. Also, the velocity fields obtained from KD simulations have been previously validated against flow fields measured experimentally *in vitro* using phase-contrast MRI [48]. We express the RAD equations as

$$\begin{array}{c} L\left(A_{i}\right)=R\left(A_{i}\right)=\sum_{k}R_{i}^{k}\;, \end{array}$$ (7)

where $R_{i}^{k}$ represents the different contributions to production or depletion of *A*_*i*_.

The definition of the RAD equation, for each of the chemical species considered in our model is presented below:

Resting Platelets:
  $$\begin{array}{c} L\left[RP\right]=-k_{AP}^{AP}\left[AP\right]\left[RP\right]-k_{AP}^{II_{\alpha }}\left[RP\right]-A\left(SR\right)\left[RP\right]\;, \end{array}$$ (8)
  where A(SR) is the activation function defined in Eq 4.
Active Platelets:
  $$\begin{array}{c} L\left[AP\right]=k_{AP}^{AP}\left[AP\right]\left[RP\right]+k_{AP}^{II_{\alpha }}\left[RP\right]+A\left(SR\right)\left[RP\right]\;. \end{array}$$ (9)
ADP:
  $$\begin{array}{c} L\left[ADP\right]={\left[ADP\right]}_{rel}L\left[AP\right]\;. \end{array}$$ (10)
Polyphosphate (PolyP):
  $$\begin{array}{c} L\left[PolyP\right]={\left[PolyP\right]}_{rel}L\left[AP\right]\;, \end{array}$$ (11)
  where [*PolyP*]_*rel*_ and [*ADP*]_*rel*_ account for ADP and PolyP released from dense granules upon platelet activation.

### Boundary conditions and initial conditions

A Dirichlet boundary condition was imposed at the inlet of the domain (proximal segment of the coronary artery) to match the circulating concentration of platelets and other chemical species considered in our model. A no flux Neumann boundary condition was prescribed at the walls. The 3D mesh was initialized to the average circulating values of resting platelets, activated platelets, ADP, and PolyP according to literature data (Table 1). No abnormal platelet count or hematocrit is usually reported in patients with KD, therefore we assumed average population values for platelet count (2.5 10^11^ platelets/l) with 5% of activated platelets circulating in the bloodstream [49]. Chemical reaction rates were set to literature values, $k_{AP}^{AP}=3x10^{8}M^{-1}s^{-1}$[14], $k_{AP}^{SR}=0.6$ *s*^−1^[31]. Platelet activation via thrombin is neglected ($k_{AP}^{II_{\alpha }}∼0$) since we are considering thrombus initiation before thrombin production. The diffusivity constant for platelets considering the average shear rate levels observed in our hemodynamic simulations was on the order of 10^−7^. This value was estimated by combining Brownian diffusion and enhanced-shear diffusion using the definition given by Anand et al. [31]. Enhanced-shear diffusion was also considered for ADP and PolyP. Diffusion coefficients and initial conditions for the simulation are summarized in Table 1.

**Table 1:** Initial conditions and diffusion coefficient values for Reaction-Advection-Diffusion (RAD) equations.

Species	Initial concentration [nM]	Diffusion Coefficient [cm^2^/s]	Reference
RP	10	10^−7^	[14], [31]
AP	0.5	10^−7^	[49], [31]
ADP	250	25.7⋅10^−7^	[50]
PolyP	0	10^−7^	[8, 51, 52, 31]

RP = resting platelets, AP = activated platelets, ADP = adenosine diphosphate, PolyP = polyphosphate

### Biochemical platelet activation

One of the primary mediators of biochemical platelet activation is the release of chemical agonists from platelet dense granules upon activation. We assumed the following values for the contents of platelet dense granules based on the literature [7]: [*ADP*]_*content*_ = 3 *mol*/10^17^ *plt* and [*PolyP*]_*content*_ = 0.743 *mol*/10^17^ *plt*. Assuming at least 75% percent of the platelets dense granules content is released, we can estimate the corresponding concentrations of released [*ADP*]_*rel*_ and [*PolyP*]_*rel*_:

$$\begin{array}{c} \begin{array}{c} {\left[ADP\right]}_{rel}=\\ 0.75\cdot \frac{3\;mol}{10^{17}\;plt}\cdot \frac{2.5\cdot 10^{11}\;plt/l}{10\;mol/l}=562.5\;, \end{array} \end{array}$$ (12)

$$\begin{array}{c} \begin{array}{c} {\left[PolyP\right]}_{rel}=\\ 0.75\cdot \frac{0.74\;mol}{10^{17}\;plts}\frac{2.5\cdot 10^{11}\;plt/l}{10\;mol/l}=138.75\;. \end{array} \end{array}$$ (13)

Although reaction constant rates are given in terms of [*AP*] and [*RP*] we included an activation function that limits the activity of this reaction based on a minimum ADP concentration, [*ADP*] > 0.2 *μ*M, which is reported in the literature as the threshold value for reversible changes in platelets [45]. The activation function is defined as a ramp function between [*ADP*] = 0.2 *μ*M (reversible activation) and [*ADP*] = 1 *μ*M (irreversible activation).


$$\begin{array}{c} f\left(\left[ADP\right]\right)=\{\begin{array}{cc} 0 & [ADP]\leq 0.2\mu M\\ \frac{[ADP]-0.2}{0.8} & 0.2\mu M<[ADP]\leq 1\mu M\\ 1 & 1\mu M<[ADP]. \end{array} \end{array}$$ (14)

### Post-processing and quantities of interest

We performed a qualitative comparison of the distribution of [*AP*] in a normal coronary tree, and low and high thrombosis risk cases, and quantified the concentration of resting and activated platelets within the aneurysms. In addition, we quantified the concentration of agonists such as ADP and PolyP to determine if their concentration reached the critical values associated with irreversible changes in platelets and platelet aggregation in the case of ADP, and activation of factor XII (intrinsic coagulation pathway) in the case of PolyP. Critical values were set to [*ADP*]_*crit*_ = 1*μM* [*PolyP*]_*crit*_ = 2*μgr* / *ml* according to experimental values reported in the literature [8, 45]. To quantify the potential for thrombus initiation and the pro-coagulant environment induced by the aneurysm, we considered the following quantities of interest, based on concentration fields obtained from the RAD simulations:

- Volume fraction of the domain where the concentration of activated platelets is greater than or equal to a specified level of platelet activation. We define *AP*_*x*_ as the volume fraction of the aneurysm where platelet activation is greater or equal to x% activation, with full platelet activation at [*AP*] ≥ 10nM.
- Volume fraction of the aneurysm where [*ADP*] and [*PolyP*] are above the specified critical thresholds.

For patients with more than one consecutive aneurysms with no normal coronary segment in between, consecutive aneurysms were considered in aggregate.

## Results

### High concentration of activated platelets correlates with regions prone to thrombosis

**Fig 4:**
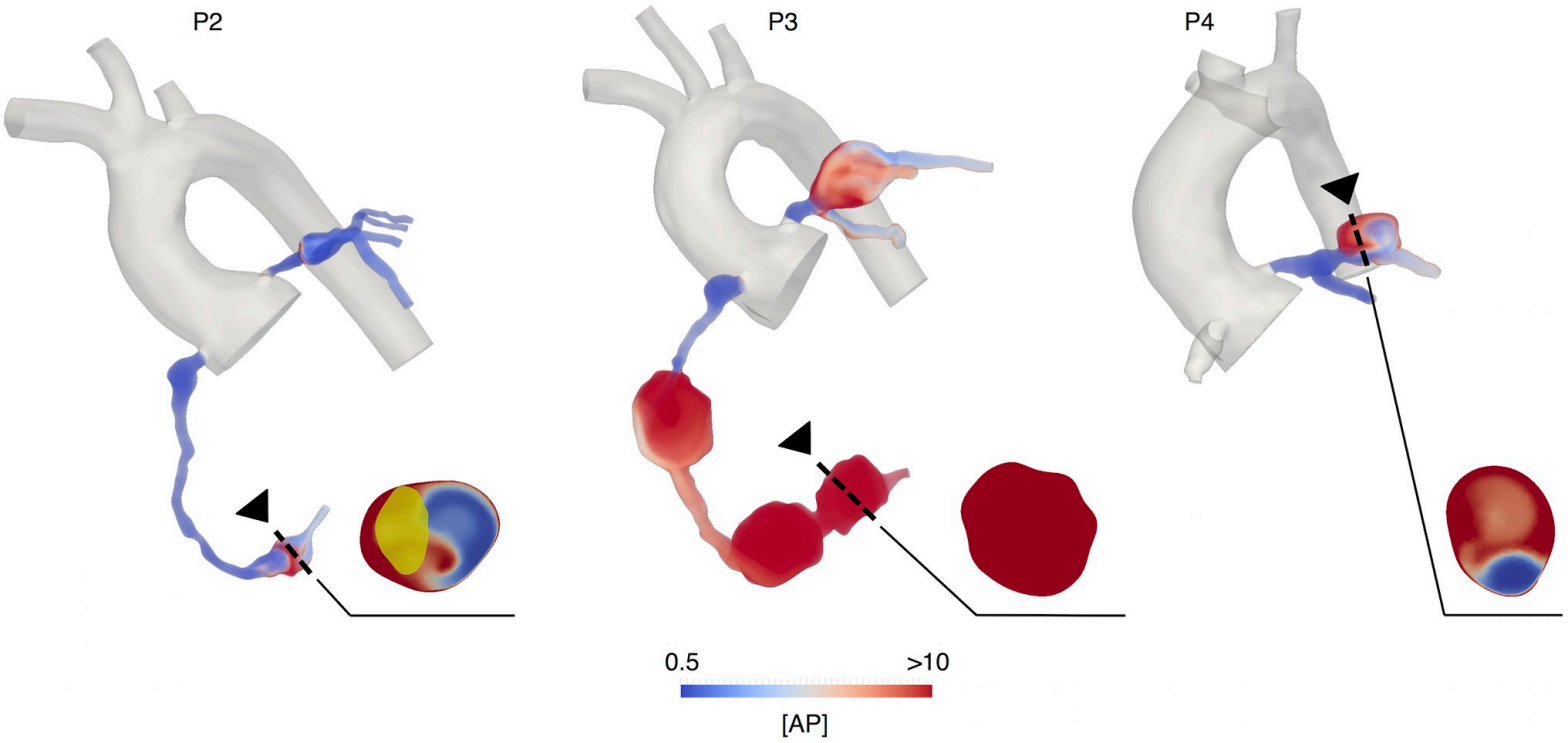
Qualitative comparison of [*AP*] spatial distribution for Kawasaki disease patients classified as high thrombotic risk. Arrow indicates region of confirmed thrombosis. Yellow shaded area represents the thrombus segmentation from image data. [*AP*] = Activated platelet concentration.

**Fig 5:**
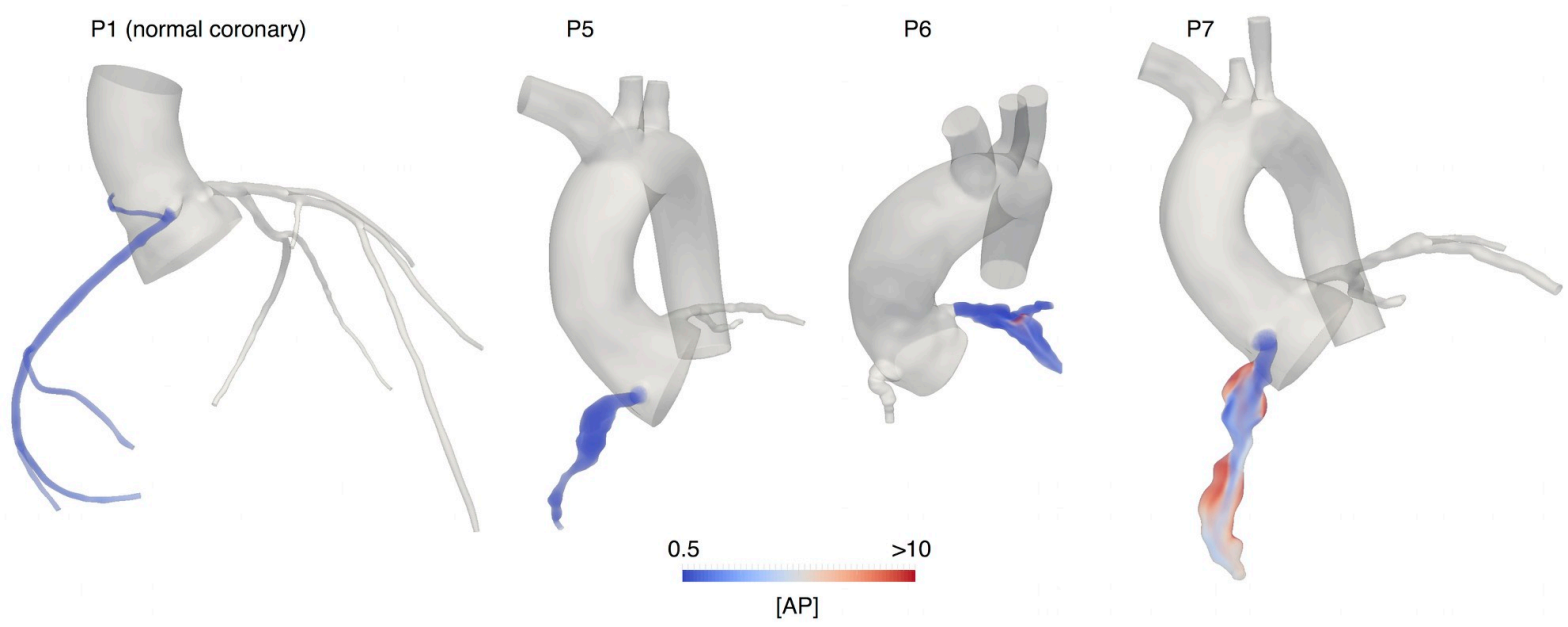
Qualitative comparison of [*AP*] spatial distribution for Kawasaki disease patients classified as low thrombotic risk and a normal coronary artery control. [*AP*] = Activated platelet concentration.

**Fig 6:**
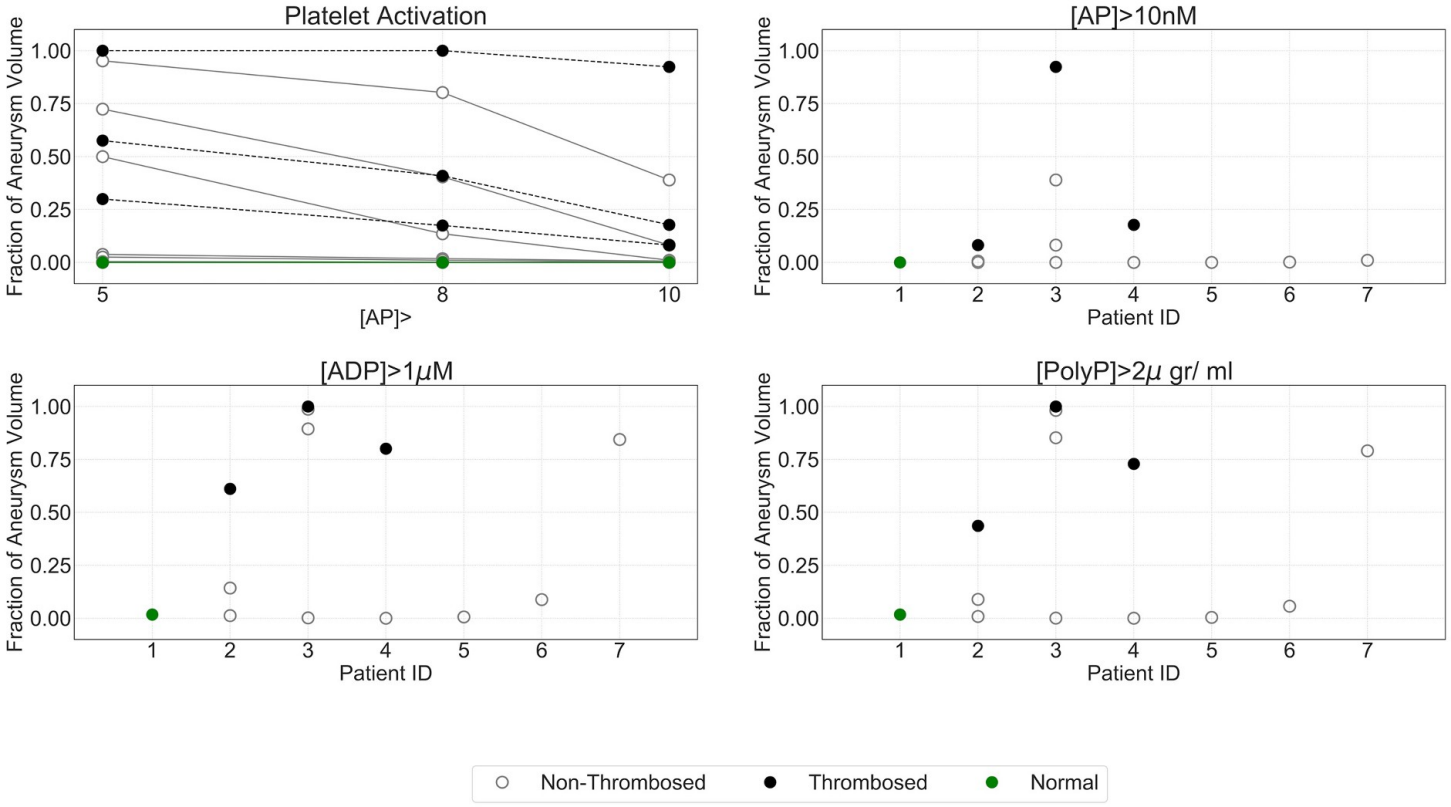
Pro-coagulant environment quantification based on concentration of activated platelets and agonists. a) Changes in fraction of aneurysm volume where [*AP*] >5, 8, 10 nM; b) fraction of aneurysm volume where [*AP*] >10 nM. Pro-coagulant environment quantification based on ADP and PolyP critical thresholds. c) Fraction of aneurysm volume where [*ADP*] > [*ADP*]_*crit*_; d) fraction of aneurysm volume where [*PolyP*] > [*PolyP*]_*crit*_. Th = confirmed thrombosis, NonTh = non confirmed thrombosis, [AP] = activated platelet concentration.

Qualitative comparison of [*AP*] spatial distribution between coronary arteries with aneurysms and the control case showed that high [*AP*] correlates with regions of confirmed thrombosis (Figs 4 and 5). In the case of patient P2, we were able to perform a direct validation of our predictions since the thrombus was still visible on the image data (Cardiac MRI). Volumetric segmentation of the clot overlapped with the area of the highest [*AP*] (Fig 4). The concentration of activated platelets in most of the aneurysms classified as high risk of thrombosis reached values substantially higher than in the normal coronary case, where [*AP*] remained close to the initial value for the simulation, which assumed a 5% platelet activation. Coronary arteries with aneurysms classified as low risk for thrombosis, presented with [*AP*] generally lower than the cases classified as high risk of thrombosis and confirmed clot formation. In patients with confirmed thrombosis, levels of [*AP*] were equal or greater than the initial concentration of non-activated platelets (10 nM/l), at the specific locations where thrombosis was reported, which implies full platelet activation. The volume fraction of the aneurysm where [*AP*] > 10 nM, representing full platelet activation, ranged from 10–100% for the high thrombotic risk patients. On the low-risk cases, this volume fraction was smaller, ranging from 0–10% (Fig 6). Volume fraction where [*AP*] >10M for the normal coronary case was 0%.

### ADP and PolyP concentration reaches critical thresholds in areas of confirmed thrombosis

Quantification of aneurysm volume fraction where ADP and PolyP were greater or equal to [*ADP*]_*crit*_ and [*PolyP*]_*crit*_ respectively suggested a more pro-coagulant environment in those aneurysms with confirmed thrombosis (Fig 6). Except for patient P7, pro-coagulant volume fractions measured were almost zero for non-thrombosis branches. Note that for patient P3 the aneurysm in series with the aneurysms where the clot was reported also exhibits larger volume fraction of pro-coagulant environment measured by [*ADP*] and [*PolyP*].

### Biochemical platelet activation is dominant in regions of flow stagnation

**Fig 7:**
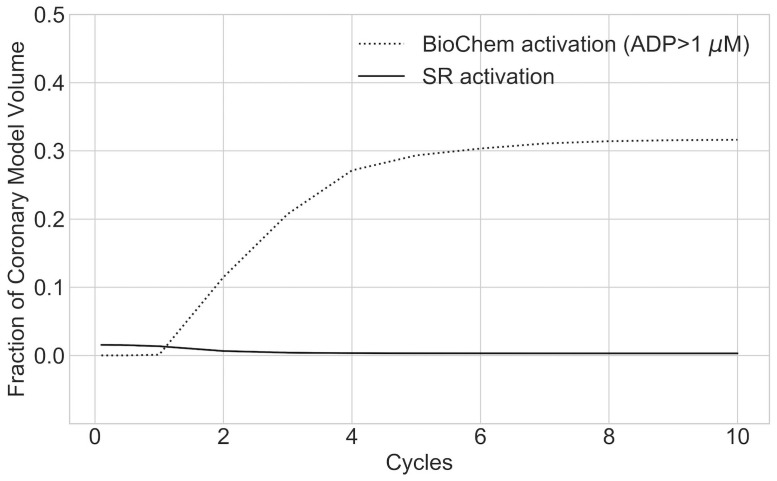
Temporal evolution of the contribution of mechanical and biochemical factors to platelet activation. Contribution to platelet activation as defined in Eq 9. Volume fraction where SR is dominant is negligible in regions of flow stagnation. Data corresponding to P2 right coronary artery. BioChem = Platelet activation due to concentration of agonists, SR = platelet activation based on cumulative shear rate activation function (Eq 4).

**Fig 8:**
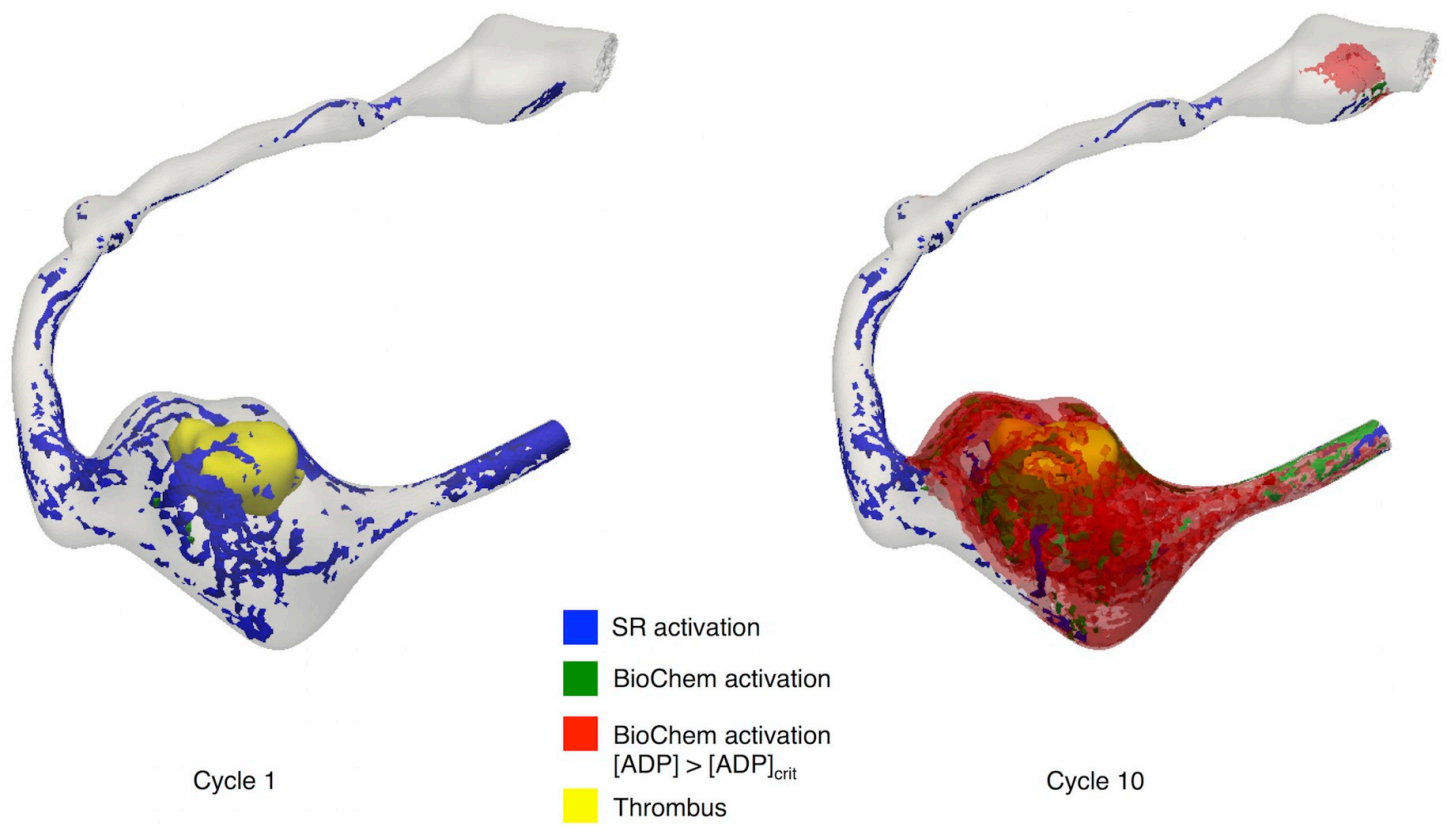
Qualitative comparison of platelet activation due to mechanical and biochemical contributions as a function of time. Thrombus 3D segmentation in yellow.

We compared the contributions of mechanical and biochemical mechanisms to platelet activation. We observed that in regions of flow stagnation, the dominant reaction term represented the contribution due to local [*AP*]. The analysis of the temporal evolution of these contributions showed that, even if at the beginning of the simulation, mechanical activation, based on cumulative shear rate, was the main driver for platelet activation, as [*AP*] increases, the biochemical activation term becomes dominant (Fig 7). The contribution of the cumulative shear rate in the cases we analyzed, even if dominant during the first or second cardiac cycles, was small. This suggests that the contribution of shear rate to platelet activation in KD aneurysms is almost negligible, and platelet activation is instead mediated by the local increase of chemical agonist concentrations in the aneurysm as a result of flow stagnation (Fig 8).

### Pro-coagulant environment quantification shows correlation with hemodynamic variables

We observed a qualitative correlation between areas of low shear stress and high residence time with regions of increased pro-coagulant activity, measured by [*AP*], [*ADP*] and [*PolyP*]. Quantitative correlation of AP_10_ with hemodynamic variables showed high correlation with residence time (r^2^ = 0.97). On the other hand, we observed low correlation of AP_10_ (volume fraction) with low wall shear stress exposure, A_*WSS*1_ (surface area fraction, TAWSS < 1 dyne/cm^2^, r^2^ = 0.23).

## Discussion

This paper is the first computational study to consider the biochemical aspects of thrombosis for a patient-specific quantification of aneurysm pro-coagulant environment in KD. The model is also novel in the sense that it investigates the conditions for thrombus initiation without relying on an extrinsic trigger to the coagulation cascade. This model can be extended to investigate other cardiovascular problems, such as aortic dissection, that present with stagnation-driven thrombus as a result of complex flow patterns and without a focal injury site. Also, the timeline for clot formation may be distinct from other coagulation and thrombosis scenarios where the clot forms in seconds or hours, usually as a result of an extrinsic component such as TF being released into the bloodstream, or the exposure of sub-endothelial collagen and vWF. The model presented here relies solely on the anatomy of the patient, at a given point in time, and the physics and biochemistry that govern blood flow and platelet activation to predict *where* and *if* the clot is likely to form.

Previous studies have demonstrated that hemodynamics, in particular, wall shear stress and residence time correlate with regions prone to thrombosis [3, 5]. Here we provided added value to residence time calculation since we not only identify areas of potential accumulation of platelets and agonists, but also we quantify the local concentration of chemical agonists and compare with critical thresholds related to platelet aggregation in the case of ADP, and factor XII activation in the case of PolyP.

Our results show qualitatively high correlation between regions of low WSS and high residence time and regions with pro-coagulant activity. However, surface metrics (area exposed to low WSS, A_*WSS*1_) are not well correlated with volume metrics (volume fraction where AP concentration is high, AP_10_). The fraction of surface area exposed to low WSS (A_*WSS*1_) is calculated with a threshold of TAWSS < 1 dynes/cm^2^. This suggests that wall quantities such as low WSS exposure (A_*WSS*1_) may not be enough to quantify the abnormal hemodynamics and pro-coagulant environment in this case. Even if the fraction of the wall exposed to pathological WSS is smaller, there could still be areas of high recirculation and flow stagnation within the aneurysms. The focus of many studies on wall quantities in relation to thrombosis is typically motivated by the assumed thrombogenic potential of the aneurysm wall and the hypothesis that platelets concentrate near the wall. The latter is referred to as platelet margination, affected by the Fåhraeus-Lindqvist effect [53]. However, limitations arise when applying this assumption in large arteries in the cardiovascular system, since the scales at which this effect has been usually observed are on the order of *μ*m which is several orders of magnitude below the typical size of coronary aneurysms. Although in some cases platelet margination has been observed at the mm scale, the development of near the wall excess of platelets required ideal flow conditions and lengths well beyond the typical coronary artery length [54].

Importantly, the margination effect has been mostly observed under ideal or healthy flow conditions, in which the flow is primarily unidirectional. Therefore it may not be straightforward to extend this behavior to situations of complex flow. Some studies have suggested shear stress levels may impact platelet margination. In particular, low wall shear stress may limit the margination effect by slowing down margination and increasing the margination length [55–59]. Therefore, based on these data, we did not expect significant margination effect in this study due to the low shear rates observed in KD aneurysms (100 s^−1^) from our hemodynamic simulations and the typical length of coronary arteries. In addition, geometry changes such as expansions or constrictions have been reported to significantly effect particle margination [60]. For these reasons, here we considered the full 3D fluid domain in our calculations of platelet activation and platelet accumulation. We observed areas of high platelet concentrations growing into the lumen of the artery in those aneurysms exhibiting exceptionally complex flow patterns. This could explain the low correlation between A_*WSS*1_ and the pro-coagulant volume fraction AP_10_ and demonstrates the added value of combining wall-surface and volumetric metrics. Since, the aneurysmal wall is abnormal in KD patients, especially after remodeling has occurred [61, 62], it is possible that the probability of platelet adhesion to the wall is higher than in normal coronary arteries. Histology and Optical Coherence Tomography studies have reported intimal hyperplasia and irreversible damage to the aneurysm wall [61, 63], however, the relationship between this damage and subsequent thrombus formation remains incompletely understood. Experimental animal models that replicate aneurysm formation *in vivo* could be useful to guide more realistic modeling of KD thrombus progression in future work.

Since we considered a linear approximation to compute the cumulative shear rate, we expected that it would increase linearly with the distance along the vessel. This holds for the normal coronary case, however, the diseased anatomy shows non-linear concentrations of higher cumulative shear rate. This is a manifestation of the complex flow patterns created in the pathological anatomy. Of note, in the diseased coronary arteries (Figs 3 and 4) we observe areas of high cumulative shear rate in both proximal and distal regions of the coronary artery. This demonstrates that the cumulative shear rate encodes information of high shear rate magnitudes as well as high exposure times, to moderate or even normal shear rates.

Our simulation results show that the contribution of shear rate to platelet activation is almost negligible for the cases considered in this study. However, calculated cumulative shear rate distributions suggest that stenosis proximal to the aneurysm may substantially increase the contribution of shear rate to platelet activation. The biochemical activation term is dominant in areas of high flow stagnation, where the accumulation of circulating pro-coagulants results in concentrations that may be sufficient to induce platelet aggregation or initiate the intrinsic coagulation cascade via factor XII activation [8]. In the absence of flow stagnation, the accumulation of activated platelets, and the increase of [*ADP*] and [*PolyP*] in the coronary models analyzed was negligible. We observed that although higher cumulative shear rate correlated with thrombus location to some extent (Fig 2), such values of cumulative shear rate were not enough to result in substantial platelet activation according to the shear-induced platelet activation function (Eq 5). To assess sensitivity of our results to changes in the activation function definition (Eq 5), we also considered a lower threshold for cumulative shear rate activation (*SR*_*cum*_ > 250) and our results did not differ substantially. Completely removing the mechanical contribution to platelet activation also did not substantially change the result. This was expected since the dominant contribution in the cases analyzed here was due to agonist-induced platelet activation.

Shear stress levels in localized areas of high activated platelet accumulation were typically < 1 dyne/cm^2^. According to experimental assessments of thrombus deformation and growth at different shear rates [37], this mechanical environment would facilitate clot growth and would induce small deformations. Future studies that include platelet dynamics similar to Lu et al. [38] would provide more insight into the potential risk of growth to total occlusion.

In our computational experiments, we did not find substantial differences between [*AP*] distribution from patient-specific velocity fields obtained from rigid wall or fluid-solid interaction simulations. However, we observed that as the flow becomes more complex, especially in the presence of large flow stagnation areas, wall deformation may have a more significant effect on near-wall transport. This should be considered in future studies, in particular, if large wall deformations are expected, such as in aortic dissection.

The low velocities and low shear rates typically observed in KD aneurysms suggest diffusion transport may have an important role. However, we did not find significant differences in the total volume of full platelet activation (*AP*_10_) comparing advection only vs. advection and diffusion solutions from our simulations. This was also confirmed by calculating the Peclet number. Considering coronary artery diameter as the characteristic length scale, Peclet number was >>1 for all cases, demonstrating that our models, at a macroscopic scale, are advection dominated. To investigate the ratio of advection to diffusion, we re-computed the Peclet number using the size of platelets as the characteristic length, approximating flow moving through an aggregate of platelets where the size of the opening is approximately the platelet size. Using this characteristic dimension, representative of the flow at the microscale, there are small regions near the aneurysm wall where diffusion may dominate. To capture diffusion of chemical agonists appropriately, an adaptive meshing method, which refines the mesh as platelets begin aggregating, could be integrated into the solver. In this case, the already low velocities observed in some regions of the aneurysms may be reduced substantially, increasing the diffusion contribution to the transport of agonists at this location.

We performed a pro-coagulant environment quantification as a post-processing step of patient-specific flow simulations. It is important to note that high concentration of activated platelets contributes to thrombus initiation, however this criterion alone is likely not a sufficient condition for thrombus development. For this reason, we also quantify other agonists related to thrombosis (ADP, PolyP). Future extensions of this model will consider the full multi-scale process of clot formation to assess how concentration of activated platelets and agonists directly affect this process. Because of the short time scale considered, it is reasonable to assume that the effect of the fluid-thrombus interaction at this point is very small. Therefore this data provides an initial condition for a future multi-scale thrombus growth simulation identifying regions likely to develop thrombosis due to induced pro-coagulant environments. Our analysis showed that a simulation time of 10 cardiac cycles was sufficient to capture activated platelet and agonist concentration distributions from a given patient-specific velocity field. Future studies should consider a coupled approach that solves the fluid and the RAD equations simultaneously and that accounts for the changes in the flow field as the clot grows. Multi-phase models such as those proposed by Xu et al. [35] and Zheng et al. [40] could potentially be adapted for patient-specific simulations. Experimental data describing clot composition and material properties would be key to increase the realism of these models and would allow validation of the simulated clot formation process under flow.

Our model simulations did not include the full intrinsic coagulation cascade, but the estimation of the critical conditions that may trigger the intrinsic coagulation pathway ([*PolyP*] > [*PolyP*]_*crit*_). Empirical PolyP biochemical data including activation rate constant for factor XII activation needs to be incorporated to achieve a patient-specific intrinsic coagulation cascade model from patient-specific anatomy to clot formation. In addition, future studies aiming to simulate thrombus growth and concentrated on the microscale region near the wall would need to consider a more precise diffusivity value for PolyP, which is specific for PolyP chains released upon platelet activation.

## Conclusion

This computational modeling study illustrates possible mechanisms for thrombus initiation under low shear conditions, such as those induced by aneurysms. In the absence of extremely high shear rates, prolonged exposure to moderate shear rates has the potential to activate platelets. In the normal coronary case we observe that even if platelets are activated due to high cumulative shear rates, the activated platelets and chemical agonists released due to platelet activation are typically washed out of the domain after one cardiac cycle. That is, platelets do not accumulate anywhere in the coronary model. On the contrary, in the case of aneurysms, we observe a significant accumulation of both activated platelets and chemical agonists due to flow stagnation. Furthermore, the local concentration of agonists in some aneurysms reaches levels comparable to those reported as critical thresholds to induce platelet activation, amplifying the effect of shear rate activation and creating an environment prone to thrombus initiation, potentially though the intrinsic coagulation pathway. Current guidelines rely only on the diameter of the aneurysm as the criterion for initiating systemic anticoagulation. Our results suggest that it is precisely for those patients, such as patient P2, who are near the cutoff Z-score value that alternative metrics, based on hemodynamic and biochemical simulations, such as pro-coagulant environment volume quantification, can add value to thrombotic risk prediction. In addition, we showed that coronary artery aneurysm thrombosis may depend to a great degree on platelet activation, suggesting some type of antiplatelet therapy as the most efficient antithrombotic therapy.

This work provides a new framework to investigate thrombus initiation from a patient-specific perspective. The model presented here could help identify regions at higher risk of thrombosis based on patient-specific hemodynamics, even in the absence of a focal lesion, as well as the best strategies for thrombosis prevention by analyzing the most significant contributions to platelet activation in each case. Results presented here would motivate prospective studies of larger patient cohorts. Future work should include information on patient-specific coagulation profiles to increase patient-specificity and clinical relevance. This approach could also enable the addition of anticoagulation drugs into the model to evaluate their efficiency and dose management, and design treatment strategies tailored for each patient.

## References

[1] McCrindle BW, Rowley AH, Newburger JW, Burns JC, Bolger AF, Gewitz M, et al. Diagnosis, treatment, and long-term management of Kawasaki disease: a scientific statement for health professionals from the American Heart Association. Circulation. 2017;135(17):e927–e999. doi: 10.1161/CIR.0000000000000484 28356445

[2] Payne RM, Burns KM, Glatz AC, Li D, Li X, Monagle P, et al. A multi-national trial of a direct oral anticoagulant in children with cardiac disease: Design and rationale of the Safety of ApiXaban On Pediatric Heart disease On the preventioN of Embolism (SAXOPHONE) study. American Heart Journal. 2019;217:52–63. doi: 10.1016/j.ahj.2019.08.002 31493728 PMC6861679

[3] Grande Gutierrez N, Mathew M, McCrindle BW, Tran JS, Kahn AM, Burns JC, et al. Hemodynamic variables in aneurysms are associated with thrombotic risk in children with Kawasaki disease. International journal of cardiology. 2019;281:15–21. doi: 10.1016/j.ijcard.2019.01.092 30728104 PMC6511338

[4] Grande Gutierrez N, Kahn A, Burns JC, Marsden AL. Computational blood flow simulations in Kawasaki disease patients: Insight into coronary artery aneurysm hemodynamics. Global cardiology science & practice. 2017;2017(3). 10.21542/gcsp.2017.29 PMC5856960 29564350

[5] Sengupta D, Kahn AM, Kung E, Moghadam ME, Shirinsky O, Lyskina GA, et al. Thrombotic risk stratification using computational modeling in patients with coronary artery aneurysms following Kawasaki disease. Biomechanics and modeling in mechanobiology. 2014;13(6):1261–1276. doi: 10.1007/s10237-014-0570-z 24722951 PMC4990134

[6] Sengupta D, Kahn AM, Burns JC, Sankaran S, Shadden SC, et al. Image-based modeling of hemodynamics in coronary artery aneurysms caused by Kawasaki disease. Biomechanics and modeling in mechanobiology. 2012;11(6):915–932. doi: 10.1007/s10237-011-0361-8 22120599 PMC6091534

[7] Ruiz FA, Lea CR, Oldfield E, Docampo R. Human platelet dense granules contain polyphosphate and are similar to acidocalcisomes of bacteria and unicellular eukaryotes. Journal of Biological Chemistry. 2004;279(43):44250–44257. doi: 10.1074/jbc.M406261200 15308650

[8] Müller F, Mutch NJ, Schenk WA, Smith SA, Esterl L, Spronk HM, et al. Platelet polyphosphates are proinflammatory and procoagulant mediators in vivo. Cell. 2009;139(6):1143–1156. doi: 10.1016/j.cell.2009.11.001 20005807 PMC2796262

[9] Smith SA, Mutch NJ, Baskar D, Rohloff P, Docampo R, et al. Polyphosphate modulates blood coagulation and fibrinolysis. Proceedings of the National Academy of Sciences. 2006;103(4):903–908. doi: 10.1073/pnas.0507195103 16410357 PMC1347979

[10] Choi SH, Smith SA, Morrissey JH. Polyphosphate is a cofactor for the activation of factor XI by thrombin. Blood. 2011;118(26):6963–6970. doi: 10.1182/blood-2011-07-368811 21976677 PMC3245215

[11] Zhu S, Travers RJ, Morrissey JH, Diamond SL. FXIa and platelet polyphosphate as therapeutic targets during human blood clotting on collagen/tissue factor surfaces under flow. Blood. 2015;126(12):1494–1502. doi: 10.1182/blood-2015-04-641472 26136249 PMC4573872

[12] Ruggeri ZM, Orje JN, Habermann R, Federici AB, Reininger AJ. Activation-independent platelet adhesion and aggregation under elevated shear stress. Blood. 2006;108(6):1903–1910. doi: 10.1182/blood-2006-04-011551 16772609 PMC1895550

[13] Fogelson AL. Continuum models of platelet aggregation: formulation and mechanical properties. SIAM Journal on Applied Mathematics. 1992;52(4):1089–1110. doi: 10.1137/0152064

[14] Kuharsky AL, Fogelson AL. Surface-mediated control of blood coagulation: the role of binding site densities and platelet deposition. Biophysical journal. 2001;80(3):1050–1074. doi: 10.1016/S0006-3495(01)76085-7 11222273 PMC1301304

[15] Fogelson AL, Neeves KB. Fluid mechanics of blood clot formation. Annual review of fluid mechanics. 2015;47:377–403. doi: 10.1146/annurev-fluid-010814-014513 26236058 PMC4519838

[16] Leiderman K, Fogelson AL. Grow with the flow: a spatial–temporal model of platelet deposition and blood coagulation under flow. Mathematical medicine and biology: a journal of the IMA. 2011;28(1):47–84. 20439306 10.1093/imammb/dqq005 PMC3499081

[17] Leiderman K, Fogelson AL. The influence of hindered transport on the development of platelet thrombi under flow. Bulletin of mathematical biology. 2013;75(8):1255–1283. doi: 10.1007/s11538-012-9784-3 23097125 PMC6097848

[18] Brass LF, Diamond SL. Transport physics and biorheology in the setting of hemostasis and thrombosis. Journal of Thrombosis and Haemostasis. 2016;14(5):906–917. doi: 10.1111/jth.13280 26848552 PMC4870125

[19] Kim OV, Xu Z, Rosen ED, Alber MS. Fibrin networks regulate protein transport during thrombus development. PLoS computational biology. 2013;9(6):e1003095. doi: 10.1371/journal.pcbi.1003095 23785270 PMC3681659

[20] Chatterjee MS, Denney WS, Jing H, Diamond SL. Systems biology of coagulation initiation: kinetics of thrombin generation in resting and activated human blood. PLoS computational biology. 2010;6(9):e1000950. doi: 10.1371/journal.pcbi.1000950 20941387 PMC2947981

[21] Bluestein D, Niu L, Schoephoerster R, Dewanjee M. Steady flow in an aneurysm model: correlation between fluid dynamics and blood platelet deposition. Journal of biomechanical engineering. 1996;118(3):280–286. doi: 10.1115/1.2796008 8872248

[22] Liu ZL, Ku DN, Aidun CK. Mechanobiology of shear-induced platelet aggregation leading to occlusive arterial thrombosis: A multiscale in silico analysis. Journal of Biomechanics. 2021;120:110349. doi: 10.1016/j.jbiomech.2021.110349 33711601

[23] Bluestein D, Niu L, Schoephoerster RT, Dewanjee MK. Fluid mechanics of arterial stenosis: relationship to the development of mural thrombus. Annals of biomedical engineering. 1997;25(2):344. doi: 10.1007/BF02648048 9084839

[24] Nobili M, Sheriff J, Morbiducci U, Redaelli A, Bluestein D. Platelet activation due to hemodynamic shear stresses: damage accumulation model and comparison to in vitro measurements. ASAIO journal (American Society for Artificial Internal Organs: 1992). 2008;54(1):64. doi: 10.1097/MAT.0b013e31815d6898 18204318 PMC2756061

[25] Shadden SC, Hendabadi S. Potential fluid mechanic pathways of platelet activation. Biomechanics and modeling in mechanobiology. 2013;12(3):467–474. doi: 10.1007/s10237-012-0417-4 22782543 PMC3526694

[26] Hansen KB, Arzani A, Shadden SC. Mechanical platelet activation potential in abdominal aortic aneurysms. Journal of biomechanical engineering. 2015;137(4):041005. doi: 10.1115/1.4029580 25588057 PMC4321116

[27] Nesbitt WS, Westein E, Tovar-Lopez FJ, Tolouei E, Mitchell A, Fu J, et al. A shear gradient–dependent platelet aggregation mechanism drives thrombus formation. Nature medicine. 2009;15(6):665. doi: 10.1038/nm.1955 19465929

[28] Menichini C, Cheng Z, Gibbs RG, Xu XY. Predicting false lumen thrombosis in patient-specific models of aortic dissection. Journal of The Royal Society Interface. 2016;13(124):20160759. doi: 10.1098/rsif.2016.0759 27807275 PMC5134025

[29] Taylor JO, Meyer RS, Deutsch S, Manning KB. Development of a computational model for macroscopic predictions of device-induced thrombosis. Biomechanics and modeling in mechanobiology. 2016;15(6):1713–1731. doi: 10.1007/s10237-016-0793-2 27169403

[30] Sheriff J, Soares JS, Xenos M, Jesty J, Bluestein D. Evaluation of shear-induced platelet activation models under constant and dynamic shear stress loading conditions relevant to devices. Annals of biomedical engineering. 2013;41(6):1279–1296. doi: 10.1007/s10439-013-0758-x 23400312 PMC3640664

[31] Anand M, Rajagopal K, Rajagopal K. A model incorporating some of the mechanical and biochemical factors underlying clot formation and dissolution in flowing blood. Computational and mathematical methods in medicine. 2003;5(3-4):183–218.

[32] Di Achille P, Tellides G, Figueroa C, Humphrey J. A haemodynamic predictor of intraluminal thrombus formation in abdominal aortic aneurysms. Proceedings of the Royal Society A: Mathematical, Physical and Engineering Sciences. 2014;470(2172):20140163. doi: 10.1098/rspa.2014.0163

[33] Yazdani A, Li H, Humphrey JD, Karniadakis GE. A general shear-dependent model for thrombus formation. PLoS computational biology. 2017;13(1):e1005291. doi: 10.1371/journal.pcbi.1005291 28095402 PMC5240924

[34] Xu Z, Chen N, Shadden SC, Marsden JE, Kamocka MM, Rosen ED, et al. Study of blood flow impact on growth of thrombi using a multiscale model. Soft Matter. 2009;5(4):769–779. doi: 10.1039/B812429A

[35] Xu Z, Chen N, Kamocka MM, Rosen ED, Alber M. A multiscale model of thrombus development. Journal of the Royal Society Interface. 2007;5(24):705–722. doi: 10.1098/rsif.2007.1202 PMC2607450 17925274

[36] Xu Z, Lioi J, Mu J, Kamocka MM, Liu X, Chen DZ, et al. A multiscale model of venous thrombus formation with surface-mediated control of blood coagulation cascade. Biophysical journal. 2010;98(9):1723–1732. doi: 10.1016/j.bpj.2009.12.4331 20441735 PMC2862154

[37] Xu S, Xu Z, Kim OV, Litvinov RI, Weisel JW, et al. Model predictions of deformation, embolization and permeability of partially obstructive blood clots under variable shear flow. Journal of the Royal Society Interface. 2017;14(136):20170441. doi: 10.1098/rsif.2017.0441 29142014 PMC5721151

[38] Lu Y, Lee MY, Zhu S, Sinno T, Diamond SL. Multiscale simulation of thrombus growth and vessel occlusion triggered by collagen/tissue factor using a data-driven model of combinatorial platelet signalling. Mathematical medicine and biology: a journal of the IMA. 2017;34(4):523–546. 27672182 10.1093/imammb/dqw015 PMC5798174

[39] Menichini C, Xu XY. Mathematical modeling of thrombus formation in idealized models of aortic dissection: initial findings and potential applications. Journal of mathematical biology. 2016;73(5):1205–1226. doi: 10.1007/s00285-016-0986-4 27007280 PMC5055578

[40] Zheng X, Yazdani A, Li H, Humphrey JD, Karniadakis GE. A three-dimensional phase-field model for multiscale modeling of thrombus biomechanics in blood vessels. PLoS computational biology. 2020;16(4):e1007709. doi: 10.1371/journal.pcbi.1007709 32343724 PMC7224566

[41] Yazdani A, Li H, Bersi MR, Di Achille P, Insley J, Humphrey JD, et al. Data-driven modeling of hemodynamics and its role on thrombus size and shape in aortic dissections. Scientific reports. 2018;8(1):2515. doi: 10.1038/s41598-018-20603-x 29410467 PMC5802786

[42] Di Achille P, Tellides G, Humphrey J. Hemodynamics-driven deposition of intraluminal thrombus in abdominal aortic aneurysms. International journal for numerical methods in biomedical engineering. 2017;33(5):e2828. 10.1002/cnm.2828 PMC5332472 27569676

[43] Arzani A, Suh GY, Dalman RL, Shadden SC. A longitudinal comparison of hemodynamics and intraluminal thrombus deposition in abdominal aortic aneurysms. American Journal of Physiology-Heart and Circulatory Physiology. 2014;307(12):H1786–H1795. doi: 10.1152/ajpheart.00461.2014 25326533 PMC4269702

[44] Li H, Sampani K, Zheng X, Papageorgiou DP, Yazdani A, Bernabeu MO, et al. Predictive modelling of thrombus formation in diabetic retinal microaneurysms. Royal Society Open Science. 2020;7(8):201102. doi: 10.1098/rsos.201102 32968536 PMC7481715

[45] Hubbell JA, McIntire LV. Platelet active concentration profiles near growing thrombi. A mathematical consideration. Biophysical journal. 1986;50(5):937–945. doi: 10.1016/S0006-3495(86)83535-4 3790695 PMC1329819

[46] Bazilevs Y, Calo VM, Tezduyar TE, Hughes TJ. YZ*β* discontinuity capturing for advection-dominated processes with application to arterial drug delivery. International Journal for Numerical Methods in Fluids. 2007;54(6-8):593–608. doi: 10.1002/fld.1484

[47] Updegrove A, Wilson NM, Merkow J, Lan H, Marsden AL, et al. SimVascular: an open source pipeline for cardiovascular simulation. Annals of biomedical engineering. 2017;45(3):525–541. doi: 10.1007/s10439-016-1762-8 27933407 PMC6546171

[48] Kung E, Kahn AM, Burns JC, Marsden A. In vitro validation of patient-specific hemodynamic simulations in coronary aneurysms caused by Kawasaki disease. Cardiovascular engineering and technology. 2014;5(2):189–201. doi: 10.1007/s13239-014-0184-8 25050140 PMC4103185

[49] Sorensen EN, Burgreen GW, Wagner WR, Antaki JF. Computational simulation of platelet deposition and activation: I. Model development and properties. Annals of biomedical engineering. 1999;27(4):436–448. doi: 10.1114/1.201 10468228

[50] Kiviniemi TO, Yegutkin GG, Toikka J, Paul S, Aittokallio T, Janatuinen T, et al. Pravastatin-induced improvement in coronary reactivity and circulating ATP and ADP levels in young adults with type 1 diabetes. Frontiers in physiology. 2012;3:338. doi: 10.3389/fphys.2012.00338 22934084 PMC3429103

[51] Yeon JH, Mazinani N, Schlappi TS, Chan KY, Baylis JR, Smith SA, et al. Localization of short-chain polyphosphate enhances its ability to clot flowing blood plasma. Scientific reports. 2017;7(1):1–10. doi: 10.1038/srep42119 28186112 PMC5301195

[52] Zheng G, Price WS. Environmental NMR: diffusion ordered spectroscopy methods. eMagRes. 2007; p. 561–574.

[53] Fahraeus R, Lindqvist T. The viscosity of the blood in narrow capillary tubes. American Journal of Physiology-Legacy Content. 1931;96(3):562–568. doi: 10.1152/ajplegacy.1931.96.3.562

[54] Aarts P, Van Den Broek S, Prins GW, Kuiken G, Sixma JJ, et al. Blood platelets are concentrated near the wall and red blood cells, in the center in flowing blood. Arteriosclerosis: An Official Journal of the American Heart Association, Inc. 1988;8(6):819–824. doi: 10.1161/01.ATV.8.6.819 3196226

[55] Eckstein EC, Bilsker DL, Waters CM, Kippenhan JS, Tilles AW. Transport of platelets in flowing blood. Annals of the New York academy of sciences. 1987;516:442–452. doi: 10.1111/j.1749-6632.1987.tb33065.x 3439741

[56] Tilles AW, Eckstein EC. The near-wall excess of platelet-sized particles in blood flow: its dependence on hematocrit and wall shear rate. Microvascular research. 1987;33(2):211–223. doi: 10.1016/0026-2862(87)90018-5 3587076

[57] Crowl L, Fogelson AL. Analysis of mechanisms for platelet near-wall excess under arterial blood flow conditions. Journal of fluid mechanics. 2011;676:348. doi: 10.1017/jfm.2011.54

[58] Mehrabadi M, Ku DN, Aidun CK. Effects of shear rate, confinement, and particle parameters on margination in blood flow. Physical Review E. 2016;93(2):023109. doi: 10.1103/PhysRevE.93.023109 26986415

[59] Reasor DA, Mehrabadi M, Ku DN, Aidun CK. Determination of critical parameters in platelet margination. Annals of biomedical engineering. 2013;41(2):238–249. doi: 10.1007/s10439-012-0648-7 22965639

[60] Carboni EJ, Bognet BH, Cowles DB, Ma AW. The Margination of Particles in Areas of Constricted Blood Flow. Biophysical journal. 2018;114(9):2221–2230. doi: 10.1016/j.bpj.2018.04.010 29742415 PMC5961517

[61] Dionne A, Ibrahim R, Gebhard C, Bakloul M, Selly JB, Leye M, et al. Coronary wall structural changes in patients with Kawasaki disease: new insights from optical coherence tomography (OCT). Journal of the American Heart Association. 2015;4(5):e001939. doi: 10.1161/JAHA.115.001939 25991013 PMC4599424

[62] Dionne A, Ibrahim R, Gebhard C, Benovoy M, Leye M, Déry J, et al. Difference between persistent aneurysm, regressed aneurysm, and coronary dilation in Kawasaki disease: an Optical Coherence Tomography Study. Canadian Journal of Cardiology. 2018;34(9):1120–1128. doi: 10.1016/j.cjca.2018.05.021 30093299

[63] Orenstein JM, Shulman ST, Fox LM, Baker SC, Takahashi M, Bhatti TR, et al. Three linked vasculopathic processes characterize Kawasaki disease: a light and transmission electron microscopic study. PloS one. 2012;7(6):e38998. doi: 10.1371/journal.pone.0038998 22723916 PMC3377625

